# Unveiling the Masquerader: A Rare Case of Pigmented Erythroplasia of Queyrat With Successful Multimodal Treatment

**DOI:** 10.7759/cureus.84749

**Published:** 2025-05-24

**Authors:** Mouna Guechchati, Zakia Douhi, Jaafar Marrakchi Benjaafar, Sara Elloudi, FatimaZahra Mernissi

**Affiliations:** 1 Dermatology, Hassan II University Hospital, Fez, MAR; 2 Urology, Hassan II University Hospital, Fez, MAR

**Keywords:** dermoscopy, genital pigmented lesions, penile carcinoma in situ, photodynamic therapy, pigmented erythroplasia of queyrat

## Abstract

Pigmented erythroplasia of Queyrat (EQ) is a rare variant of in situ squamous cell carcinoma that can clinically and dermoscopically mimic melanoma, which makes it hard to diagnose. We report the case of a 66-year-old man with a slowly progressive pigmented penile plaque. Dermoscopy and histology confirmed the diagnosis of pigmented EQ. The patient was successfully treated with photodynamic therapy followed by reconstructive surgery. This case highlights the importance of considering pigmented EQ in the differential diagnosis of genital pigmented lesions and illustrates the effectiveness of a conservative multimodal approach.

## Introduction

Erythroplasia of Queyrat (EQ) is a rare carcinoma in situ of the glans penis and prepuce, representing an in situ form of squamous cell carcinoma. It accounts for a subset of penile cancers, which are predominantly squamous in origin (>95%) [1]. EQ typically affects mucosal or transitional surfaces, including both the glans penis and the prepuce, and often presents as a well-demarcated erythematous plaque. While the classic presentation is non-pigmented, a pigmented variant can occur, posing a significant diagnostic challenge due to its resemblance to other pigmented lesions, including melanoma and pigmented Bowen’s disease. This diagnostic ambiguity emphasizes the need for histological confirmation and careful dermoscopic evaluation. Herein, we describe a rare case of pigmented EQ managed successfully with photodynamic therapy (PDT) followed by reconstructive surgery, underlining the importance of a multimodal therapeutic approach in achieving both oncologic control and functional preservation.

## Case presentation

This report describes the case of a 66-year-old male diagnosed with pigmented erythroplasia of Queyrat, successfully managed with a combined therapeutic approach.

**Figure 1:**
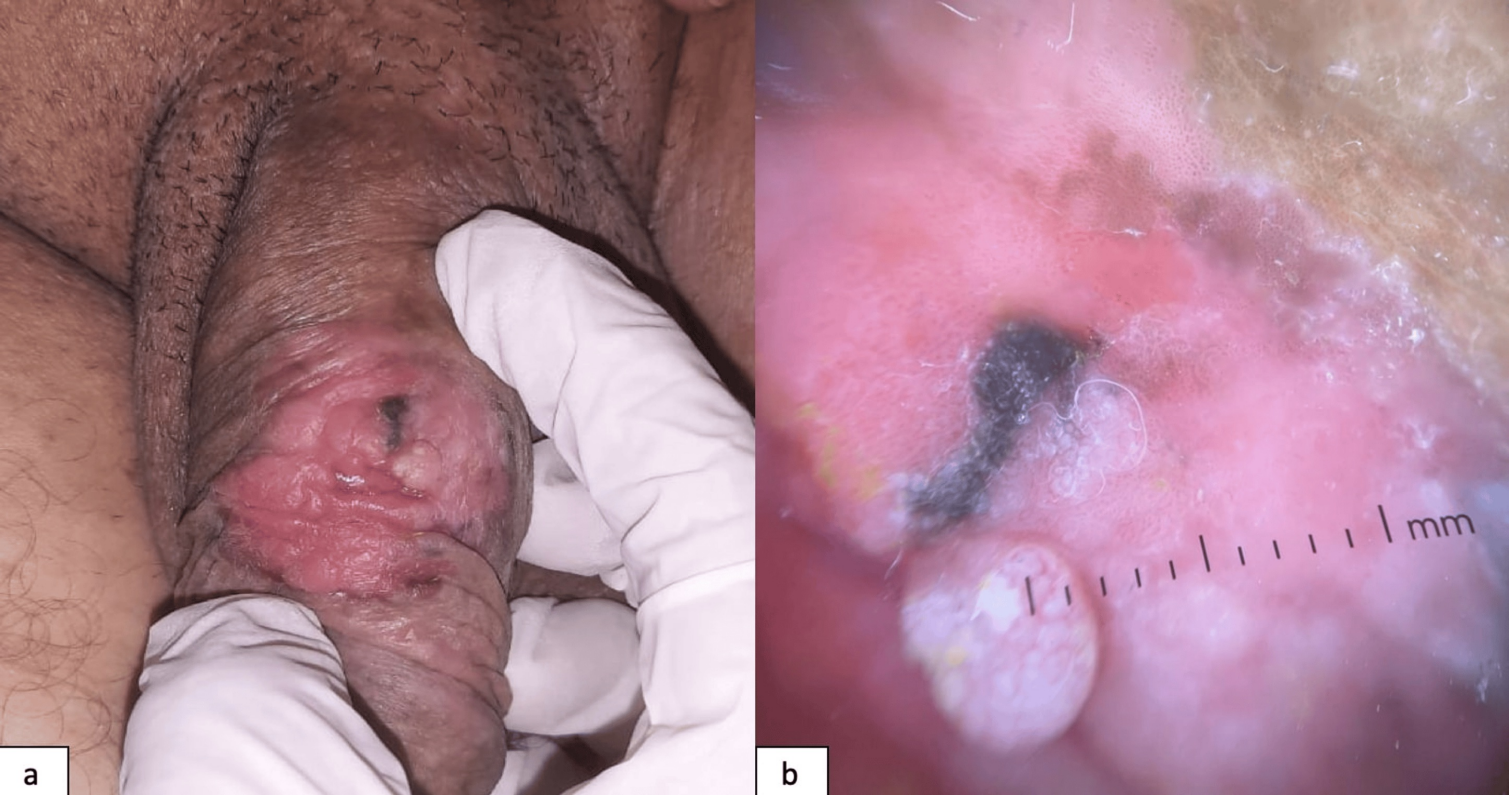
(a) Clinical presentation: Pigmented erythematous plaque of approximately 5 cm on the penile shaft, with a central dark pigmentation and palpable induration. (b) Dermoscopic features: Dermoscopy showing dotted and glomerular vessels, whitish structureless areas, and central black pigmentation suggestive of pigmented Bowen’s disease

The patient reported a history of syphilis, for which he had received treatment, with a favorable course according to his account. He subsequently noted the appearance of a painless penile lesion, which had been progressively evolving over approximately three years. Dermatological examination revealed a well-circumscribed 5 cm erythematous plaque on the penile shaft, featuring central black pigmentation and associated with palpable induration (Figure 1a).

**Figure 2:**
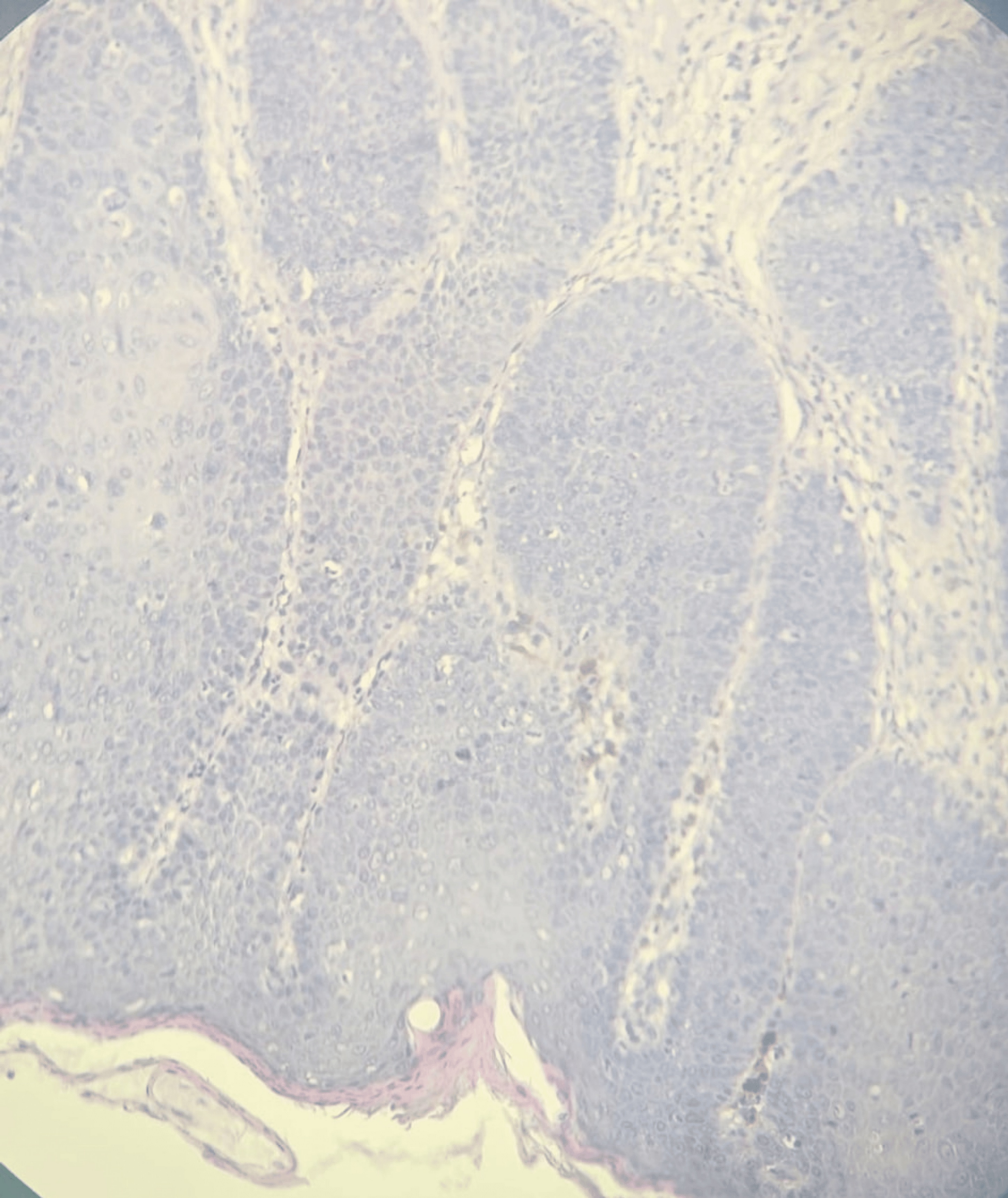
Hematoxylin and eosin stain showing disorganized atypical keratinocytes with pleomorphic hyperchromatic nuclei, pigment incontinence, and melanophages in the superficial dermis, consistent with pigmented erythroplasia of Queyrat.

Dermoscopy showed dotted and glomerular vascular structures, whitish areas, and central blackish pigmentation within the plaque (Figure 1b). A biopsy was taken from both the pigmented and atypically vascularized areas. Histopathological analysis showed a disorganized proliferation of atypical keratinocytes with deeply stained pleomorphic nuclei. This was associated with a moderately dense lymphocytic infiltrate in the superficial dermis, pigmentary incontinence, and numerous melanophages (macrophages that have engulfed melanin pigment), without evidence of dermal invasion (Figure 2). Histopathological analysis supported the diagnosis of pigmented EQ.

Ultrasound of the inguinal lymph nodes revealed heterogeneous adenopathies. The patient received topical photodynamic therapy (PDT) using the methyl ester of 5-aminolaevulinic acid (MAL), which resulted in significant clinical improvement. Reconstructive plastic surgery was performed 10 months later. The patient remains under ongoing clinical and dermoscopic surveillance.

## Discussion

Erythroplasia of Queyrat (EQ) and Bowen’s disease are both forms of squamous cell carcinoma in situ, distinguished primarily by their anatomical locations. EQ typically arises on mucosal surfaces, such as the glans penis, whereas Bowen’s disease occurs on keratinized skin. Despite these differences in presentation, both conditions share similar histopathological features and clinical courses [2].

EQ typically affects the glans penis, foreskin, or urethral meatus in older adult males; most commonly those over the age of 50, and presents as a well-defined, velvety, bright red plaque. The exact etiology remains unknown [1].

The pigmented variant accounts for only about 6% of all Bowen's disease lesions and is rarely found on genital skin in white populations. Pigmented Bowen's disease (pBD) is known as a clinical mimic of a broad range of skin tumors [3].

An alternative hypothesis proposes that pigmentation in anogenital EQ may arise from local thermal effects or physiological hyperpigmentation. Pigmented Bowen's disease (pBD) may also develop over pre-existing pigmented lesions such as melanotic macules or seborrheic keratoses [3]. This hypothesis should be further investigated and supported by larger case series or molecular studies.

The dermoscopic features of BD were first described in 2004 by Argenziano et al., who highlighted glomerular vessels as a suggestive sign [4]. More recent studies describe structureless hypopigmented areas, brown or gray dots arranged linearly, and linearly arranged glomerular vessels as characteristic features of pigmented Bowen’s disease [3].

Dermoscopic features characteristic of EQ, as reported in the literature, include structureless pink zones, clustered vessels, either dotted or glomerular in shape, and focal erosions [5]. All of these features were observed in our patient. However, because melanoma is a well-known clinical mimic and dermoscopic data on anogenital melanomas are limited, histopathological confirmation is recommended prior to initiating any topical or destructive therapies [3].

Management options for penile EQ include local excision, laser therapy, photodynamic therapy, and topical treatments such as 5-fluorouracil or 5% imiquimod cream. Penile-preserving strategies are particularly encouraged for small or localized lesions to maintain both penile function and sexual quality of life. In our case, initial treatment with PDT yielded a favorable clinical response, allowing for a conservative surgical approach.

The patient subsequently underwent reconstructive surgery with favorable clinical progress to date, underscoring the value of a multimodal and stepwise therapeutic strategy.

Close follow-up is essential to detect early recurrence and to support adherence to follow-up visits, prescribed treatments, and recommended hygienic measures [1].

## Conclusions

Diagnosing pigmented erythroplasia of Queyrat (EQ) remains challenging due to its mimicry of other pigmented tumors, including melanoma and pigmented Bowen’s disease. It should always be considered in the differential diagnosis of atypical pigmented lesions. Dermoscopy is a valuable, non-invasive diagnostic tool that enhances clinical accuracy, supports therapeutic decision-making, and facilitates longitudinal monitoring. In this case, it played a central role in the initial diagnostic approach, evaluation of PDT response, and postoperative surveillance.
